# Current Progress in Vaccines against Merkel Cell Carcinoma: A Narrative Review and Update

**DOI:** 10.3390/vaccines12050533

**Published:** 2024-05-13

**Authors:** Thilo Gambichler, David Schrama, Riina Käpynen, Sera S. Weyer-Fahlbusch, Jürgen C. Becker, Laura Susok, Florian Kreppel, Nessr Abu Rached

**Affiliations:** 1 Department of Dermatology, Ruhr-University Bochum, 44791 Bochum, Germany; riina.kaeypnen@klinikum-bochum.de (R.K.); nessr.aburached@kklbo.de (N.A.R.); 2 Department of Dermatology, Dortmund Hospital gGmbH and Faculty of Health, Witten-Herdecke University, 44122 Dortmund, Germany; serasalina.weyer-fahlbusch@klinikumdo.de (S.S.W.-F.); laura.susok@klinikumdo.de (L.S.); 3 Department of Dermatology and Phlebology, Christian Hospital Unna, 59423 Unna, Germany; 4 Department of Dermatology, Venereology and Allergology, University Hospital Würzburg, 97080 Würzburg, Germany; schrama_d@ukw.de; 5 Translational Skin Cancer Research, DKTK Partner Site Essen/Düsseldorf, West German Cancer Center, Department of Dermatology, University Duisburg-Essen, 45122 Essen, Germany; j.becker@dkfz.heidelberg.de; 6 German Cancer Research Center (DKFZ), 69120 Heidelberg, Germany; 7 Chair of Biochemistry and Molecular Medicine, Center for Biomedical Education and Research (ZBAF), Witten/Herdecke University, 58453 Witten, Germany; florian.kreppel@uni-wh.de

**Keywords:** skin cancer, vaccines, oncolytic virus, mRNA, personalized, peptides, immunology, nanoparticles

## Abstract

Merkel cell carcinoma is a rare, aggressive skin cancer that mainly occurs in elderly and immunocompromised patients. Due to the success of immune checkpoint inhibition in MCC, the importance of immunotherapy and vaccines in MCC has increased in recent years. In this article, we aim to present the current progress and perspectives in the development of vaccines for this disease. Here, we summarize and discuss the current literature and ongoing clinical trials investigating vaccines against MCC. We identified 10 articles through a PubMed search investigating a vaccine against MCC. From the international clinical trial database Clinical.Trials.gov, we identified nine studies on vaccines for the management of MCC, of which seven are actively recruiting. Most of the identified studies investigating a vaccine against MCC are preclinical or phase 1/2 trials. The vaccine principles mainly included DNA- and (synthetic) peptide-based vaccines, but RNA-based vaccines, oncolytic viruses, and the combination of vaccines and immunotherapy are also under investigation for the treatment of MCC. Although the management of MCC is changing, when compared to times before the approval of immune checkpoint inhibitors, it will still take some time before the first MCC vaccine is ready for approval.

## 1. Introduction

### 1.1. Vaccines in General

Vaccines ideally work in the prevention of infections (e.g., polio, smallpox, diphtheria, tuberculosis), when a person is administered the vaccine before being infected. In the case of malignancies, however, the situation is more complex, since only a minor fraction of malignancies are associated with infectious agents [1]. However, de Martel et al. [2] recently reported that, for 2018, about 2.2 million infection-attributable cancer cases were diagnosed worldwide, corresponding to an infection-attributable age-standardized incidence rate of 25 cases per 100,000 person-years [2]. Cancers that are caused by viral infection include cervical, liver, and head and neck malignancies, and their prevention by vaccines plays a crucial role in reducing the risk of cancer development. Consequently, vaccines have been developed and approved, for example, that are capable of preventing hepatitis B virus or human papilloma virus (HPV) infection and thus protecting against the development of the aforementioned virus-related cancers [1]. In this setting, when cancer has already developed, it is possible to identify targets for malignant cells that can aid in distinguishing tumor cells from healthy cells [3,4,5,6,7,8,9,10,11,12,13]. Especially interesting are tumor entities for which most patients express shared antigens. This is, for example, the case in virus-driven tumors like Merkel cell carcinoma (MCC). In this concise review, we aim to present the current progress and future challenges in vaccine development against MCC [14,15,16,17,18,19,20,21,22,23,24,25,26,27,28,29,30,31,32,33,34,35,36,37,38,39,40,41,42,43,44,45,46,47,48,49,50,51,52,53,54,55,56,57,58,59].

#### 1.1.1. Vaccine Principles in Oncology

As mentioned above, prophylactic vaccines have been designed to combat tumor development by reducing malignancy-causing infections, whereas therapeutic vaccination aims at inducing strong host immune responses and immunological memory, in order to effectively kill existing tumor cells [14]. Notably, most cancer vaccine studies in the last century demonstrated variable, mostly disappointing, efficacy. Nevertheless, the exceptional successes observed in some cases undoubtedly confirm that therapeutic vaccinations can induce durable remissions [15]. 

The most commonly employed strategy for the development of therapeutic tumor vaccination banks on the formation of tumor-specific cytotoxic CD8-positive T lymphocytes, because of their unique capability to eradicate tumor cells due to specific cell recognition [16,17]. Furthermore, antibody-mediated cytotoxic mechanisms may significantly contribute to control cancer growth and be exploited by cancer vaccines [16,17,18]. In particular, an antibody that specifically binds to tumor cells is able to trigger their eradication by antibody-induced cellular cytotoxicity, antibody-induced cellular phagocytosis, and complement-dependent cytotoxicity [16,18]. So far, however, the latter mechanisms have been commonly utilized through passive immunotherapy of malignancies via the intravenous application of therapeutic antibodies in patients, rather than humoral-based tumor vaccination that aims to induce endogenous host anti-cancer antibody responses [16,18,19]. In addition, the anti-tumor adaptive immunological responses induced by anti-tumor vaccines can be further enhanced by coactivation of innate immunity. For instance, lymphoid cells of the innate immune system, including natural killer (NK) cells or invariant NK cells, have the capability to control tumor cells in a complementary manner to cytotoxic T lymphocytes [16,17,18,19,20].

Despite comprehensive research, however, only a few vaccines have made it into oncology to date: (i) two prophylactic vaccines against HPV and the hepatitis B virus, preventing the development of virus-associated cervical cancer and hepatocellular carcinoma, respectively [21,22]; and (ii) three FDA-approved therapeutic vaccines, i.e., bacillus Calmette–Guerin vaccine for treatment of non-muscle-invasive bladder cancer, Sipuleucel-T (the only FDA-approved autologous cellular immunotherapy for metastatic castration-resistant prostate cancer), and talimogene laherparepvec “Imlygic^®^”, an oncolytic viral drug for treatment of advanced melanoma [16,17,23]. To date, melanoma represents the only cutaneous malignancy for which significant research in the field of vaccinology has been conducted [16,18,19,20]. Certainly, immunotherapy in solid tumors is generally hampered by inefficient drug delivery to the tumor cells. Notably, nanoparticles flow to different regions of tumors via blood vessels and must then cross the vessel wall, and finally penetrate through the interstitial space to reach the target cells. The abnormal organization and structure of the tumor vasculature leads to tortuous and leaky vessels and heterogeneous blood flow [24]. Furthermore, the lack of functional lymphatic vessels and the vascular hyperpermeability inside the tumors leads to interstitial hypertension [24]. Hence, delivering tumor vaccines to the draining lymph nodes to improve immunosurveillance and limit systemic tolerance plays an important role in oncologic immunotherapy. Therefore, there are multiple nanomedical vaccine principles including peptide- and dendritic-cell-based, mRNA-based, viral-vector-based (oncolytic plasmids), oncolytic-virus-based, and exosome-based vaccines to improve drug delivery. Every strategy has its strengths and limitations [24,25,26,27,28,29,30,31,32,33,34,35,36,37].

#### 1.1.2. Peptides

Peptide-based vaccines consist of short peptide sequences derived from tumor-specific antigens. The optimal size of the nanocarriers for peptides is 15–70 nm for transportation via bloodstream and for lymphatic vessel entrance [31]. However, since peptides are weakly immunogenic, they require combination with potent immune adjuvants to enhance their immunogenicity [32]. Upon injection, these peptides are taken up by dendritic cells (DCs), i.e., professional antigen presenting cells. Maturated by the adjuvant, the DCs will migrate to the lymph node, where they present the peptides to T cells. This will activate and expand peptide-specific T cells able to fight cancer cells [30]. Peptide vaccines are relatively easy and cheap to produce, but require identifying proper adjuvants. More complicated and more expensive to manufacture are DC vaccines. In this approach, patient’s dendritic cells are collected and loaded with specific antigens in the laboratory. Similarly as for peptide vaccination, after injection or infusion, these antigen-loaded DCs migrate to the lymph nodes and trigger a specific immune response against the presented antigens. Dendritic cell vaccines are being tested as a monotherapy or in combination with checkpoint inhibitors [29]. 

#### 1.1.3. mRNA

mRNA vaccines consist of a small amount of synthetic mRNA encoding the desired antigen [28]. The limitation of mRNA vaccines is that only small amounts reach the lymph nodes, as the chemical structure of mRNA is unstable and their negative charge prohibits passive cell membrane transport [25,27]. To increase their stability, they are typically delivered in lipid-based vehicles. Since these lipid-based vehicles are associated with poor transfection efficiency and in vivo toxicity, much effort is spent on optimizing the delivery systems [25]. Nevertheless, mRNA vaccines have a good safety profile and can effectively stimulate the immune system. After injection of the mRNA vaccine, the mRNA-loaded lipid-based vehicle enters the cells via endocytosis, where the mRNA can be released into the cytoplasmic compartment and subsequently translated into protein. Upon degradation by the proteasome and loading onto MHC molecules, the peptides are displayed at the cell surface, activating specific T cells. The advantage of mRNA vaccines is rapid production, in contrast to other vaccines [26].

#### 1.1.4. Oncolytic-Virus-Based Vaccines

Not only inactivated components are used in vaccines, but also oncolytic viruses. The aim of oncolytic viruses as vaccines is to directly infect and destroy cancer cells [34]. The concept of so-called virotherapy is based on two interdependent mechanisms: the lysis of tumor cells by a replicating lytic virus (“oncolysis”), and the subsequent induction of an immune response against tumor antigens (“vaccination”). While lytic virus replication damages the tumor microenvironment and can render the tumor accessible for immune cells, the cell fragments produced can serve as novel antigenic materials to induce anti-tumor responses. [35]. Depending on the type of virus and the type of tumor, high doses and multiple administrations of oncolytic-virus-based vaccines might be required for effective therapy, particularly when the susceptibility of the tumor cells to the virus is low. This in turn can induce anti-virus immune responses, some of which appear to have adjuvant effects. However, pre-existing immunity to an oncolytic virus as a result of a natural infection earlier in a patient’s life has been shown to limit treatment efficacy. Current developments address this issue by using viruses with low seroprevalence, by employing genetic surface modifications to remove antigenic epitopes, or by shielding viruses with synthetic or natural polymers that provide a stealth cloak on the virus surface [3,6,8,34,35].

#### 1.1.5. Oncolytic Plasmids/Oncotoxic Proteins

A viral vector vaccine is a type of vaccine that employs a virus to transport genetic material, which can be translated by the recipient’s host cells into mRNA [36]. The antigen is then produced by the infected cells, leading to an immune response. A major advantage of plasmids is their decreased size in comparison to whole viruses. Diep et al. highlighted the effects of oncotoxic viral proteins on the example of apoptin, with E4orf4 and NS1 inhibiting proliferation by interfering with the p53-independent apoptosis pathway in tumor cells, while leaving healthy cells unaffected. Furthermore, oncotoxic proteins or plasmid DNA can improve enrichment of therapeutics at tumor sites and avoid excessive immune activation [37]. For example, a first clinical study of H-1PV treatment in glioma patients has yielded evidence of intratumoral synthesis of the viral oncotoxic protein NS1 and immune cell infiltration [59].

#### 1.1.6. Exosome-Based Cancer Vaccine

Extracellular vesicles have a size of 30–100 nm with a lipid bilayer membrane [38,39]. Via fusion with the cell membrane, its contents are released into the cell. In addition, exosomes carry membrane proteins to increase the immune response. T-cells are primed via exosomes comparably to T-cells being primed by dendritic cells. In contrast, exosomes can also induce immunosuppressive activity and promote inhibition of lymphocyte proliferation by increasing the suppressive effects of regulatory T-cells. Exosome based vaccines with highly expressed antigens are generated by modified tumor cells [37,40].

### 1.2. Merkel Cell Carcinoma 

Merkel cell carcinoma (MCC) is a rare type of skin cancer that mainly affects older and/or immunosuppressed individuals [41,42]. In recent decades, however, the incidence rates of MCC have ascended worldwide; for example, in the United States, from 0.15 in 1986 to 0.7/100,000 in 2016. Nations with the highest MCC incidence (expressed as cases per million, World Standard Population) in the period 2003–2007 were Australia (male: 5.2, female: 2.2), New Zealand (male: 4.5, female: 3.2), and the USA (white population, male: 4.2, female: 1.9). The risk factors for the development of MCC include male sex, age over 75 years, fair skin complexion, ultraviolet (UV) exposure, and immunosuppression. Projections propose that, due to aging populations, an increase in immunosuppressed patients, and enhanced UV exposure, MCC incidence rates will continue to rise [41]. 

Although MCC cells share morphological, immunohistological, and ultrastructural features with Merkel cells, current data point to an epithelial progenitor cell as the cell of origin or a multilinear origin, given the expression of neuroendocrine, epithelial/fibroblast, and B-lymphoid markers [10]. Interestingly, a viral- or UV-associated carcinogenesis has been identified [10]. In the Northern hemisphere, virus-positive cases prevail, with MC polyomavirus (MCPyV) clonally integrated into the tumor cells in about 80% of patients. These tumors are dependent on the expression of the viral oncoproteins, i.e., the T antigens (small T (sT) and large T (LT)). MCPyV-negative MCC is characterized by a high number of UV-induced DNA mutations, being increased 90-fold compared to MCPyV-positive tumors. Apart from RB1 and TP53 mutations, aberrations also occur frequently in Notch genes, as well as in the PI3K/AKT/mTOR signaling pathway. Although MCPyV proteins have been associated with increased genomic instability, the noticeable lack of mutations in MCPyV-positive tumors suggests that their genomes are relatively stable. In contrast, MCPyV-negative MCC is clearly driven by mutations, including many that contribute to genomic instability, as mentioned above. Hence, MCCs of both etiologies should express potential immunogenic antigens, i.e., viral peptides in the case of virus-positive MCC. In the case of MCPyV-negative MCC, neoantigens (aberrant proteins) that develop due to mutations in the tumor represent potential immunogenic agents [43]. The biological behavior of MCC is highly aggressive, with high rates of local recurrences, regional lymph node, and distant metastasis. Hence, the 5-year relative survival estimates from population-based registries (US, Netherlands, Finland, Spain, Germany, and New Zealand) range from 36% to 65% for men and for women 47% to 84% [43]. When compared to the chemotherapy era, the management of advanced MCC has been significantly improved since the introduction of immunotherapy, particularly using immune checkpoint inhibitors (ICI). Nevertheless, only about 50% of patients with MCC benefit from immunotherapy. Hence, additional or combination approaches, particularly including vaccination strategies, are highly needed to augment immune responses that target and eliminate MCC [10,11,41,42]. Given the general success of immunotherapy in MCC and the expression of a necessary viral protein shared by virus-positive cases, this tumor is a prime candidate for vaccination strategies. Consequently, the development of virus-directed vaccines against MCC has significantly increased in recent years [7,43,44,45,46,47,48,49,50,51]. For example, peptide- and DNA-based vaccines and combination therapies with vaccines and ICI are currently being investigated for treatment of MCC in preclinical and clinical trials.

## 2. Therapeutic Vaccines for MCC

### 2.1. Published Preclinical and Clinical Studies

On PubMed Central, we identified 10 published papers on preclinical and clinical studies dealing with vaccine strategies against MCC, which have been summarized in Table 1 [7,8,44,45,46,47,48,49,50,51,55]. Almost all studies summarized in Table 1 are of preclinical nature, including murine models and multiepitope computational vaccine design strategies; only three investigations had a phase I or II study design. The vaccine principles mostly included DNA- and (synthetic) peptide-based vaccines. 

Proof of concept was delivered in several reports by the group of Chien-Fu Hung. They used a mouse model based on a melanoma cell line expressing LT ectopically, since a mouse model representing human MCC has only recently been described [52]. Both prophylactic and therapeutic vaccinations with a plasmid encoding LT demonstrated high efficiency in preventing (out)growth of tumors. The anti-tumor effect triggered by vaccination was largely dependent on CD4+ T cells [50]. By using a modified DNA vaccine encoding a LT fused to calreticulin, a damage-associated molecular pattern (DAMP), they were able to elicit LT-specific CD8+ immune responses in mice [45]. In contrast, in a syngeneic mouse model with an sT-expressing melanoma cell line, vaccination with an sT-encoding plasmid was sufficient to induce sT antigenic peptide specific CD8+ T cells demonstrating antitumor efficiency [46]. 

**Figure 1:**
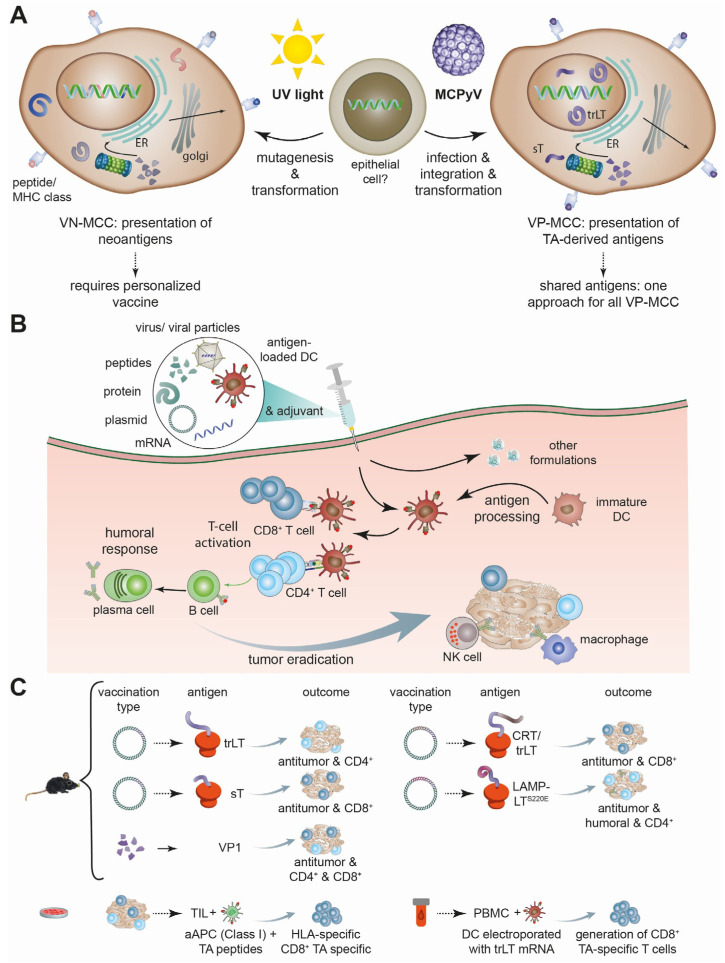
Antigenicity of MCC, vaccination principle, and MCPyV-specific preclinical vaccination approaches. (**A**) Due to their different etiologies, virus-negative (VN-) and virus-positive (VP-) MCC differ in the presentation of antigens, i.e., neoantigens and TA-derived peptides, respectively. Hence, they require different vaccination strategies. (**B**) Commonly used types of vaccination and their mechanisms. (**C**) Preclinical testing to demonstrate induction of MCPyV-specific immunity. For the mouse models, tumor cells were used that ectopically expressed the viral peptide targeted by the vaccination. aAPC: artificial antigen presenting cell; DC: endritic cell; ER: endoplasmatic reticulum; PBMC: peripheral blood mononuclear cell; TIL: tumor infiltrating lymphocytes; trLT: truncated LT (in general, only trLT is expressed in MCC).

It should be remembered that the T antigens are oncoproteins (Figure 1). Therefore, plasmids encoding them have to be used with caution. One recently published study used, as a safety measure, a plasmid encoding a mutated LT (LT^S220E^) known to have lost its pro-proliferating activity [53] fused to LAMP1. This vaccine was able to induce antigen-specific CD4 T-cell responses and a sufficiently strong humoral response to delay tumor growth in mice as a monotherapy, but also synergistically with ICI [7]. Based on these positive results, a phase I study with eight MCC patients has been completed, but the results have not yet been published (NCT05422781). A different approach to circumvent vaccinating with an oncoprotein is to use viral capsid proteins for vaccination. Indeed, a peptide-based VP1 vaccination with the crassocephalum rabens-derived adjuvant CRA could induce VP1-specific CD4+ and CD8+ immune responses mediating an anti-tumor effect [51].

An in silico peptide design study also focused on the capsid proteins VP1 and VP2, since they were identified as best candidates [48,51]. The designed vaccine should be widely applicable and induce a good immune response. For prophylactic vaccination, the disease is too rare to be cost-effective. Nevertheless, another in silico study designed a multiepitope vaccine for prophylactic vaccination [48]. Since the multiepitope vaccine also contained epitopes of sT and LT, this design could be at least partially tested as a therapeutic vaccine. 

For several different HLA haplotypes (HLA-A*24:02, HLAB*44:01, HLA-B*35:01, HLA-B*44:02) epitopes from sT and LT have been identified, allowing identifying T-antigen-specific T cells [54]. When blood samples from control (*n* = 54) and MCC patients (*n* = 49) were analyzed, T-antigen-specific T cells were only identified in MCC patients. Finally, the authors detected 11 previously unreported T-antigen-derived epitopes, supporting evidence for T-antigen-mediated MCC tumor cell recognition [54]. For vaccination strategies, it is important that T-antigen-specific T cells are also present in the tumor, and that these can be expanded. In this regard, Jing et al. [44] probed MCC tumor-infiltrating lymphocytes (TILs) using an artificial antigen-presenting cell system and confirmed T-Ag recognition with synthetic peptides in 9 of 12 MCPyV+ MCCs [44]. The reactive TILs recognized 1 to 8 MCPyV epitopes per patient. Importantly, analysis of 16 MCPyV CD8^+^ TIL epitopes and their restricting HLA alleles covered almost 100% of patients with MCPyV+ MCC, indicating that active vaccination targeting these T-antigen-specific T cells may be a suitable therapy for MCC. Moreover, the clustering of MCPyV CD8+ T cell epitopes in the region around AA 70–110 of the LT suggests that vaccines may be able to eliminate some T-antigen regions, while retaining good population coverage. Similarly, Gerer et al. showed that mRNA-electroporated dendritic cells can induce in vitro specific T-cell responses against the MCV-truncLT in PBMC from healthy subjects, as well as MCC patients [47]. The authors concluded that this is a significant step towards a dentritic-cell-based cellular immunotherapy against MCC, but ultimately clinical trials are required to investigate whether a dendritic-cell-based vaccine is an additional therapeutic option for patients with MCC. Although not specific for MCC, Bhatia et al. performed a single-arm, open label, phase-II pilot trial evaluating intratumoral delivery of IL12 plasmid DNA (tavo) using in vivo electroporation (i.t.-tavo-EP) in MCC patients (n = 15) [49]. Importantly, DNA plasmid delivery was successful, since the authors observed sustained intratumoral expression of IL12 protein along with local inflammation and an increased number of CD8+ TILs, which led to systemic immunologic and clinical responses. Within the group of patients with metastatic MCC, the observed overall response rate was 25% (3/12), with two patients showing durable clinical benefit. Among the patients with locoregional disease (n = 3), two were relapse-free at 44+ and 75+ months, respectively. The authors concluded that gene electrotransfer, specifically i.t.-tavo-EP, warrants further investigation for immunotherapy of cancer [49]. In another phase-I study, the effect of intralesional administration of IFx-Hu2.0, a plasmid encoding the immunogenic bacterial protein Emm55 was tested in advanced MCC patients resistant to ICI therapy. While the therapy by itself had as best response stable disease among five MCC and four cSCC patients, four of the MCC patients experienced objective response to ICI re-challenge, the immediate post-protocol therapy. An additional 11 patients are planned to be included in the trial (NCT04160065). 

**Table 1:** Collection of pre-clinical and clinical studies on vaccines for the treatment of Merkel cell carcinoma.

Reference/Year	Phase of Study	Vaccine Principle	Target/Effect
Zeng et al., 2012 [50]	0	DNA vaccine encoding MCPyV-LT aal-258	Syngeneic C57BL/6 mice model with B16 tumors (melanoma) expressing LT/vaccinations was efficient in prophylactic and therapeutic setting; antitumor effect in vaccinated animals was largely dependent on CD4+ T cells
Xu et al., 2021 [51]	0	VP1 peptide-based vaccine	Syngeneic BALB/c mice model with CMS-5 tumors (sarcoma) expressing VP1/ Antitumor effect of the vaccine mainly mediated by VP1-specific CD4+ and CD8+ T cell responses
Bhatia et al., 2020 [49]	II	IL12-encoding plasmid-DNA	Intra-tumoral administration of plasmid and in vivo electroporation/ IL12 promotes adaptive type I immunity and has antitumor activity (15 patients); vaccine proved to be a safe therapeutic option
Jing et al., 2020 [44]	0	In vitro stimulation of TIL	MCC TILs/TA specificity of TILs could be detected in 9 of 12 patients
Gomez et al., 2012 [45]	0	DNA vaccine encoding LT-calreticulin fusion protein	Syngeneic C57BL/6 mice model with B16 tumors expressing LT/vaccinated mice with LT-calreticulin produced more LT-specific CD8+ cells and lived longer
Gomez et al., 2013 [46]	0	DNA vaccine encoding sT	Syngeneic C57BL/6 mice model with B16 tumors expressing sT/ st-targeting vaccine resulted in prolonged survival, decreased tumor size, and increased sT-specific CD8+ cells
Gerer et al., 2017 [47]	0	truncLT-mRNA-transfected dendritic cells (DC)	PBMCs of control and MCC patients/optimized DC are able to induce MCPyV-antigen-specific immune responses in vitro in both cohort (3 of 5 MCC patients)
Almansour 2022 [8]	0	In silico multiepitope vaccine design	Chimeric multi-epitopes-based vaccine to capsid VP1 and VP2 generated in silico strong immune responses with production of interferons and cytokines
Imon et al., 2023 [48]	0	In silico multiepitope vaccine design	Vaccine candidate consisted of peptides derived from LT, sT, VP1, VP2, and VP3 antigens, and demonstrated real-life-like immune response by computer-aided immune simulation
Buchta Rosean et al., 2023 [7]	0	DNA vaccine encoding LT^S220A^-LAMP1 fusion protein	Syngeneic C57BL/6 mice model with B16 tumors expressing LT^S220A^/antigen-specific CD4+ cells and humoral response
Brohl et al., 2023 [55]	I	DNA plasmid encoding immunogenic bacterial protein Emm5	Treatment of ICI-resistant MCC by up to three intralesional injections of IFx-Hu/well tolerated at the applied doses; best response to therapy was SD, but 4 of 5 MCC patients experienced objective response to ICI re-challenge

### 2.2. Currently Registered Trials on Vaccines against Merkel Cell Carcinoma

In the international database Clinical.Trials.gov, we identified nine vaccine-based studies designed for the management of MCC (Table 2), of which seven were actively recruiting and two active but non-recruiting (estimated study completion planned for 06/24). Furthermore, four studies were found that had either been withdrawn or terminated.

All of the identified trials were found to be phase I and II (5 in phase I, 2 in phase I/II, and 2 in phase II) designed for dose escalation/dose finding and/or to investigate the objective response rate, safety, tolerability or efficacy of the vaccines being tested. The most commonly used vaccination categories currently recruiting represent the oncolytic-virus-based vaccines, including modified herpes simplex type 1 virus (NCT04349436), oncolytic adenovirus (NCT05076760), and two trials on vaccinia virus strategies (NCT04725331, NCT05859074). One personalized neoantigen-peptide-based, one plasmid-DNA-based, and one RNAi-based vaccination strategy were also found among the ongoing studies. Both of the active but non-recruiting studies focused on oncolytic herpes viruses (T-VEC), for which positive case reports for MCC patients have also been published (PMID: 35499413, 35666757, 31516997, 30450405).

Intratumoral injection was found to be the major application form of these vaccines (NCT05076760, NCT04725331, NCT06014086, NCT05859074, NCT04160065). Consequently, common eligibility criteria for patients in these studies are for their lesions to be accurately localizable, easily palpable (cutaneous, subcutaneous, or lymph node), and superficial enough to enable intralesional injection. Only one vaccine (NCT05269381) was given subcutaneously, aiming to target tumor cells systemically. In the majority of studies, treatment was given to locally advanced, unresectable, metastatic, and largely pre-treated and/or simultaneously treated (standard of care systemic treatments) solid tumors including MCC. Only one study aimed to examine the effect of neoadjuvant treatment (NCT06014086) with intra-tumoral injection following operation on the local lesions in question. Monotherapy with the tested vaccines was found in two of the active trials (NCT04160065, NCT06014086), while the majority of studies included a second study arm as combination with anti-PD-1 therapy: one nivolumab therapy (NCT05076760) and three pembrolizumab therapies (NCT04725331, NCT05859074, NCT05269381). 

## 3. Discussion

Overall, the number of vaccine trials in MCC is increasing, but they are limited to preclinical, phase I and II. Notably, although the results of preclinical testing of MCC vaccines are encouraging, current clinical trials are almost all not specific for MCC. Since phase 3 trials for vaccination studies in MCC are lacking, it will take several years before vaccines for the treatment of MCC can be licensed. However, it can be assumed that vaccines will become an important component in the treatment of skin cancers such as MCC in the future [8,9,10,11,51]. In principle, preventive vaccines as approved for cervical cancer prevention are conceivable for patients with MCPyV-positive MCCs and are under investigation in several laboratories [8,9,56]. For example, Imon et al. recently designed and identify a potential peptide vaccine candidate against MCPyV by using computational approaches that can be further utilized for subsequent vaccine construction. Their study successfully identified peptide candidates against the virus and designed a valid multiepitope vaccine construct to fight against MCPyV and boost the immune system. However, further in vitro and in vivo studies are needed in this field. Moreover, unlike HPV vaccination strategies, universal vaccination recommendations for patients with MCC are unrealistic, given the rarity of this malignancy. Nevertheless, as MCC relapses have been observed to be up to 75%, depending on disease stage, MCC vaccination may represent a valuable potential approach to reduce recurrence and mortality associated with MCC. In the case of virus-negative MCC, vaccination strategies are much more complex. However, due to the high tumor mutational burden of UV-induced virus-negative MCC and the consequently high content of neoantigens, it is likely that virus-negative MCC may also be responsive to vaccination approaches. Nevertheless, these approaches would only work on a personalized basis and are extremely expensive and time-consuming.

MCPyV-positive MCCs are prime candidates for MCC-specific vaccinations, since they express T antigens as shared immunogenic antigens. Given the positive preclinical results, it would be interesting to see those approaches tested in clinical trials. As almost all vaccine studies are directed toward MCPyV-positive MCC, there is need to develop vaccines against MCPyV-negative MCC as well. An important aspect of the efficacy of vaccines will be the development of resistance mechanisms by tumors. Under certain conditions, tumors can develop complex mechanisms to evade the immune system, so that the tumor is no longer targeted. For example, mutations in or loss of expression of the targeted protein could prevent the vaccine from working effectively against MCC. As the development, production, and delivery of cancer vaccines is currently very costly and logistically challenging, it may take longer to develop a new vaccine for such mutations or identify suitable alternative tumor antigens. Hence, RNA-based vaccines seem to be the future, as they can be developed more quickly. Indeed, during the course of the COVID-19 pandemic, mRNA-based vaccines could be adapted to various strains of SARS-CoV-2 with relative ease and in a short time period. In the further development of suitable vaccines against MCC, particular attention must also focus on the tolerability and safety of the vaccine [28,33].

A combination of immunotherapy and vaccine is a promising approach. The vaccine could prime tumor-specific T cells, while immunotherapy could boost them. This new approach could improve outcomes for patients with MCC and other skin cancers. Certainly, enhancing immune responses by combining immunotherapy and vaccines could increase the risk of serious adverse events. Potential side effects could be similar to those seen with combination immunotherapy, such as nivolumab and ipilimumab. These include autoimmune hypophysitis, thyroiditis, autoimmune colitis, and autoimmune hepatitis, particularly as we are dealing in MCC with a high-aged population [43]. Severe side effects such as cytokine release syndrome, which can be life-threatening, are also possible. On the other hand, however, directing the immune response specifically to tumor cells by vaccination might even dampen some of the toxicities observed with ICI. Nevertheless, safety aspects need to be closely monitored in clinical trials, in order to make a definitive statement. In particular, possible vaccination complications with oncological vaccines have to be discussed and monitored. Moreover, other treatment strategies against MCC and other skin cancers are currently under investigation, including PI3K/mTOR inhibitors, domatinostat, adoptive cell therapy, and next-generation immune checkpoint inhibitors, such as anti-TIM-3, anti-TIGIT, and anti-LAD-3. Other therapeutic approaches currently focus on the energy metabolism, such as glycolysis and enhanced glucose uptake, as well as decreased oxidative metabolism. Energy metabolism is altered in many cancers and may be addressed with the use of antisense oligonucleotides or GAPDH-inhibitors such as fumaric acid esters [33,57,58].

## 4. Conclusions

In conclusion, options in cancer therapy are increasing, and standard regimens are about to change. It seems very likely that novel vaccines will in the future enable personalizing the treatment of MCC, especially the virus-negative MCC. A combination of vaccine and immunotherapy represents an important component of MCC therapy in clinical trials. These trials will show whether vaccines are effective against MCC. Nevertheless, it may take some time before the first vaccine against MCC is approved. 

## Figures and Tables

**Table 2:** Current trials on therapeutic vaccines against Merkel cell carcinoma registered on Clinical.Trials.gov (accessed on 12 December 2023).

NCT/Status	Design of Study	Vaccine Category	Intervention/Treatment
NCT04160065 currently recruiting	Phase I, non-randomized, multicenter	Plasmid DNA encoding immunogenic bacterial protein Emm55	Intralesional IFx-Hu2.0 monotherapy in MCC resistant to ICI;
NCT05269381 currently recruiting	Phase I, open-label	Personalized neoantigen peptide-based	PNeoVCA: cyclophosphamide, personalized s.c. neoantigen vaccine with sargramostim, given as monotherapy or with pembrolizumab
NCT05076760 currently recruiting	Phase I, open-label	Conditionally replication-competent oncolytic adenovirus type 5 encoding transgenes for IFNβ and a recombinant chimeric form of CD40-ligand	Intratumoral injection of MEM-288 (selectively replicative in cancer cells) with and without nivolumab
NCT04349436 currently recruiting	Phase IB/II, multicenter, open-label	Oncolytic, modified herpes simplex type 1 virus (RP1)	Intra-tumoral injection of RP1
NCT04725331 currently recruiting	Phase I/II multicenter, open-label	Oncolytic vaccinia-based virus encoding CTLA4 and GM-CSF (BT-001)	Intra-tumoral BT-001 (TG6030) administered alone or in combination with pembrolizumab
NCT06014086 currently recruiting	Phase I, open Label	RNAi targeting *PD-1* (PH-762)	Neoadjuvant monotherapy using PH-762
NCT05859074 currently recruiting	Phase I, open Label	Non-replicative recombinant modified vaccinia virus Ankara encoding FLT3L and Ox40L (MQ710)	Intra-tumoral MQ710 alone or in combination with pembrolizumab
NCT02819843 active, not recruiting	Phase II open Label	Modified oncolytic herpes virus type 1 encoding GM-CSF (talimogene laherparepvec; T-VEC)	Intralesional T-VEC with or without hypofractionated radiotherapy
NCT02978625 active, not recruiting	Phase II, open Label	Modified oncolytic herpes virus type 1 encoding GM-CSF (talimogene laherparepvec; T-VEC)	Intratumoral T-VEC plus anti-PD-1 antibody (Nivolumab)

## References

[1] Montero D.A., Vidal R.M., Velasco J., Carreño L.J., Torres J.P., Benachi O M.A., Tovar-Rosero Y.-Y., Oñate A.A., O’Ryan M. (2023). Two centuries of vaccination: Historical and conceptual approach and future perspectives. Front. Public Health.

[2] de Martel C., Georges D., Bray F., Ferlay J., Clifford G.M. (2020). Global burden of cancer attributable to infections in 2018: A worldwide incidence analysis. Lancet Glob. Health.

[3] Dunn G.P., Sherpa N., Manyanga J., Johanns T.M. (2022). Considerations for personalized neoantigen vaccination in Malignant glioma. Adv. Drug Deliv. Rev..

[4] Stroffolini T., Stroffolini G. (2023). Vaccination Campaign against Hepatitis B Virus in Italy: A History of Successful Achievements. Vaccines.

[5] Pollard A.J., Bijker E.M. (2021). A guide to vaccinology: From basic principles to new developments. Nat. Rev. Immunol..

[6] Wu S.-E., Chen Y.-H., Hung C.-T., Yang B.-H. (2023). Therapeutic Cancer Vaccines for Nonmelanoma Skin Cancer. Curr. Treat. Options Oncol..

[7] Buchta Rosean C., Leyder E.C., Hamilton J., Carter J.J., Galloway D.A., Koelle D.M., Nghiem P., Heiland T. (2023). LAMP1 targeting of the large T antigen of Merkel cell polyomavirus results in potent CD4 T cell responses and tumor inhibition. Front. Immunol..

[8] Almansour N.M. (2022). Immunoinformatics- and Bioinformatics-Assisted Computational Designing of a Novel Multiepitopes Vaccine Against Cancer-Causing Merkel Cell Polyomavirus. Front. Microbiol..

[9] Joshi T.P., Farr M.A., Hsiou D.A., Nugent S., Fathy R.A., Lewis D.J. (2022). Therapeutic targets for vaccination in polyomavirus-driven Merkel cell carcinoma. Dermatol. Ther..

[10] Houben R., Celikdemir B., Kervarrec T., Schrama D. (2023). Merkel Cell Polyomavirus: Infection, Genome, Transcripts and Its Role in Development of Merkel Cell Carcinoma. Cancers.

[11] Celikdemir B., Houben R., Kervarrec T., Samimi M., Schrama D. (2023). Current and preclinical treatment options for Merkel cell carcinoma. Expert Opin. Biol. Ther..

[12] Koff W.C., Rappuoli R., Plotkin S.A. (2023). Historical Advances in Structural and Molecular Biology and How They Impacted Vaccine Development. J. Mol. Biol..

[13] Lista F., Peragallo M.S., Biselli R., de Santis R., Mariotti S., Nisini R., D’Amelio R. (2023). Have Diagnostics, Therapies, and Vaccines Made the Difference in the Pandemic Evolution of COVID-19 in Comparison with “Spanish Flu”?. Pathogens.

[14] Liu J., Fu M., Wang M., Wan D., Wei Y., Wei X. (2022). Cancer vaccines as promising immuno-therapeutics: Platforms and current progress. J. Hematol. Oncol..

[15] Coventry B.J. (2019). Therapeutic vaccination immunomodulation: Forming the basis of all cancer immunotherapy. Ther. Adv. Vaccines Immunother..

[16] Tabachnick-Cherny S., Pulliam T., Church C., Koelle D.M., Nghiem P. (2020). Polyomavirus-driven Merkel cell carcinoma: Prospects for therapeutic vaccine development. Mol. Carcinog..

[17] DeMaria P.J., Bilusic M. (2019). Cancer Vaccines. Hematol. Oncol. Clin. N. Am..

[18] Zahavi D., AlDeghaither D., O’Connell A., Weiner L.M. (2018). Enhancing antibody-dependent cell-mediated cytotoxicity: A strategy for improving antibody-based immunotherapy. Antib. Ther..

[19] Huijbers E.J.M., Griffioen A.W. (2017). The revival of cancer vaccines—The eminent need to activate humoral immunity. Hum. Vaccines Immunother..

[20] Souza-Fonseca-Guimaraes F., Cursons J., Huntington N.D. (2019). The Emergence of Natural Killer Cells as a Major Target in Cancer Immunotherapy. Trends Immunol..

[21] Mahmood F., Xu R., Awan M.U.N., Song Y., Han Q., Xia X., Wei J., Xu J., Peng J., Zhang J. (2023). HBV Vaccines: Advances and Development. Vaccines.

[22] Barra F., Leone Roberti Maggiore U., Bogani G., Ditto A., Signorelli M., Martinelli F., Chiappa V., Lorusso D., Raspagliesi F., Ferrero S. (2019). New prophylactics human papilloma virus (HPV) vaccines against cervical cancer. J. Obstet. Gynaecol..

[23] Hargrave A., Mustafa A.S., Hanif A., Tunio J.H., Hanif S.N.M. (2023). Recent Advances in Cancer Immunotherapy with a Focus on FDA-Approved Vaccines and Neoantigen-Based Vaccines. Vaccines.

[24] Jain R.K., Stylianopoulos T. (2010). Delivering nanomedicine to solid tumors. Nat. Rev. Clin. Oncol..

[25] Guevara M.L., Persano S., Persano F. (2019). Lipid-Based Vectors for Therapeutic mRNA-Based Anti-Cancer Vaccines. Curr. Pharm. Des..

[26] Fang E., Liu X., Li M., Zhang Z., Song L., Zhu B., Wu X., Liu J., Zhao D., Li Y. (2022). Advances in COVID-19 mRNA vaccine development. Signal Transduct. Target. Ther..

[27] Guo C., Manjili M.H., Subjeck J.R., Sarkar D., Fisher P.B., Wang X.-Y. (2013). Therapeutic cancer vaccines: Past, present, and future. Adv. Cancer Res..

[28] Pardi N., Hogan M.J., Porter F.W., Weissman D. (2018). mRNA vaccines—A new era in vaccinology. Nat. Rev. Drug Discov..

[29] Yu J., Sun H., Cao W., Song Y., Jiang Z. (2022). Research progress on dendritic cell vaccines in cancer immunotherapy. Exp. Hematol. Oncol..

[30] Santos P.M., Butterfield L.H. (2018). Dendritic Cell-Based Cancer Vaccines. J. Immunol..

[31] Kim S.-Y., Noh Y.-W., Kang T.H., Kim J.-E., Kim S., Um S.H., Oh D.-B., Park Y.-M., Lim Y.T. (2017). Synthetic vaccine nanoparticles target to lymph node triggering enhanced innate and adaptive antitumor immunity. Biomaterials.

[32] Malonis R.J., Lai J.R., Vergnolle O. (2020). Peptide-Based Vaccines: Current Progress and Future Challenges. Chem. Rev..

[33] Zaggana E., Konstantinou M.P., Krasagakis G.H., de Bree E., Kalpakis K., Mavroudis D., Krasagakis K. (2022). Merkel Cell Carcinoma-Update on Diagnosis, Management and Future Perspectives. Cancers.

[34] Kaufman H.L., Kohlhapp F.J., Zloza A. (2015). Oncolytic viruses: A new class of immunotherapy drugs. Nat. Rev. Drug Discov..

[35] Mondal M., Guo J., He P., Zhou D. (2020). Recent advances of oncolytic virus in cancer therapy. Hum. Vaccin. Immunother..

[36] Ura T., Okuda K., Shimada M. (2014). Developments in Viral Vector-Based Vaccines. Vaccines.

[37] Diep Y.N., Kim T.J., Cho H., Lee L.P. (2022). Nanomedicine for advanced cancer immunotherapy. J. Control. Release.

[38] Skotland T., Sandvig K., Llorente A. (2017). Lipids in exosomes: Current knowledge and the way forward. Prog. Lipid Res..

[39] Doyle L.M., Wang M.Z. (2019). Overview of Extracellular Vesicles, Their Origin, Composition, Purpose, and Methods for Exosome Isolation and Analysis. Cells.

[40] Taylor D.D., Gercel-Taylor C. (2011). Exosomes/microvesicles: Mediators of cancer-associated immunosuppressive microenvironments. Semin. Immunopathol..

[41] Silling S., Kreuter A., Gambichler T., Meyer T., Stockfleth E., Wieland U. (2022). Epidemiology of Merkel Cell Polyomavirus Infection and Merkel Cell Carcinoma. Cancers.

[42] Becker J.C., Beer A.J., DeTemple V.K., Eigentler T., Flaig M., Gambichler T., Grabbe S., Höller U., Klumpp B., Lang S. (2023). S2k Guideline—Merkel cell carcinoma (MCC, neuroendocrine carcinoma of the skin)—Update 2022. J. Dtsch. Dermatol. Ges..

[43] Becker J.C., Stang A., Schrama D., Ugurel S. (2024). Merkel Cell Carcinoma: Integrating Epidemiology, Immunology, and Therapeutic Updates. Am. J. Clin. Dermatol..

[44] Jing L., Ott M., Church C.D., Kulikauskas R.M., Ibrani D., Iyer J.G., Afanasiev O.K., Colunga A., Cook M.M., Xie H. (2020). Prevalent and Diverse Intratumoral Oncoprotein-Specific CD8+ T Cells within Polyomavirus-Driven Merkel Cell Carcinomas. Cancer Immunol. Res..

[45] Gomez B.P., Wang C., Viscidi R.P., Peng S., He L., Wu T.-C., Hung C.-F. (2012). Strategy for eliciting antigen-specific CD8+ T cell-mediated immune response against a cryptic CTL epitope of merkel cell polyomavirus large T antigen. Cell Biosci..

[46] Gomez B., He L., Tsai Y.C., Wu T.-C., Viscidi R.P., Hung C.-F. (2013). Creation of a Merkel cell polyomavirus small T antigen-expressing murine tumor model and a DNA vaccine targeting small T antigen. Cell Biosci..

[47] Gerer K.F., Erdmann M., Hadrup S.R., Lyngaa R., Martin L.-M., Voll R.E., Schuler-Thurner B., Schuler G., Schaft N., Hoyer S. (2017). Preclinical evaluation of NF-κB-triggered dendritic cells expressing the viral oncogenic driver of Merkel cell carcinoma for therapeutic vaccination. Ther. Adv. Med. Oncol..

[48] Imon R.R., Samad A., Alam R., Alsaiari A.A., Talukder M.E.K., Almehmadi M., Ahammad F., Mohammad F. (2023). Computational formulation of a multiepitope vaccine unveils an exceptional prophylactic candidate against Merkel cell polyomavirus. Front. Immunol..

[49] Bhatia S., Longino N.V., Miller N.J., Kulikauskas R., Iyer J.G., Ibrani D., Blom A., Byrd D.R., Parvathaneni U., Twitty C.G. (2020). Intratumoral Delivery of Plasmid IL12 Via Electroporation Leads to Regression of Injected and Noninjected Tumors in Merkel Cell Carcinoma. Clin. Cancer Res..

[50] Zeng Q., Gomez B.P., Viscidi R.P., Peng S., He L., Ma B., Wu T.-C., Hung C.-F. (2012). Development of a DNA vaccine targeting Merkel cell polyomavirus. Vaccine.

[51] Xu D., Jiang S., He Y., Jin X., Zhao G., Wang B. (2021). Development of a therapeutic vaccine targeting Merkel cell polyomavirus capsid protein VP1 against Merkel cell carcinoma. NPJ Vaccines.

[52] Verhaegen M.E., Harms P.W., van Goor J.J., Arche J., Patrick M.T., Wilbert D., Zabawa H., Grachtchouk M., Liu C.-J., Hu K. (2022). Direct cellular reprogramming enables development of viral T antigen-driven Merkel cell carcinoma in mice. J. Clin. Investig..

[53] Schrama D., Hesbacher S., Angermeyer S., Schlosser A., Haferkamp S., Aue A., Adam C., Weber A., Schmidt M., Houben R. (2016). Serine 220 phosphorylation of the Merkel cell polyomavirus large T antigen crucially supports growth of Merkel cell carcinoma cells. Int. J. Cancer.

[54] Hansen U.K., Lyngaa R., Ibrani D., Church C., Verhaegen M., Dlugosz A.A., Becker J.C., Straten P.T., Nghiem P., Hadrup S.R. (2022). Extended T-Cell Epitope Landscape in Merkel Cell Polyomavirus Large T and Small T Oncoproteins Identified Uniquely in Patients with Cancer. J. Investig. Dermatol..

[55] Brohl A.S., Markowitz J., Eroglu Z., Silk A.W., Hyngstrom J.R., Khushalani N.I., Tarhini A.A., De-Aquino D.B., Abraham E., Tsai K.Y. (2023). Phase 1b trial of IFx-Hu2.0, a novel personalized cancer vaccine, in checkpoint inhibitor resistant merkel cell carcinoma and cutaneous squamous cell carcinoma. J. Clin. Oncol..

[56] Boitano T.K.L., Kako T., Leath C.A. (2023). New Paradigms in the Treatment of Cervical Cancer. Obstet. Gynecol..

[57] Yurchenko K.A., Laikova K.V., Golovkin I.O., Novikov I.A., Yurchenko A.A., Makalish T.P., Oberemok V.V. (2023). Inhibitory Effect of Phosphorothioate Oligonucleotide Complementary to G6PD mRNA on Murine Melanoma. Curr. Issues Mol. Biol..

[58] Gambichler T., Majchrzak-Stiller B., Peters I., Becker J.C., Strotmann J., Abu Rached N., Müller T., Uhl W., Buchholz M., Braumann C. (2023). The effect of GP-2250 on cultured virus-negative Merkel cell carcinoma cells: Preliminary results. J. Cancer Res. Clin. Oncol..

[59] Geletneky K., Nüesch J.P., Angelova A., Kiprianova I., Rommelaere J. (2015). Double-faceted mechanism of parvoviral oncosuppression. Curr. Opin. Virol..

